# Announcements

**Published:** 2015-02-06

## Guidance Available for Implementing and Managing Contact Tracing for Ebola in Countries Without Ebola Outbreaks

CDC has posted on its website the guidance document, “CDC Methods for Implementing and Managing Contact Tracing for Ebola Virus Disease in Less-Affected Countries” (available at http://www.cdc.gov/vhf/ebola/pdf/contact-tracing-guidelines.pdf). With Ebola, the importance of contact tracing is twofold. First, closely following all contacts of an Ebola patient during the 21-day incubation period can prevent secondary transmission. Second, detection of secondary cases early in the disease course allows them to be isolated before further transmission can occur. Rigorous attention to contact tracing is a crucial step in the containment of Ebola; a single missed contact can result in ongoing transmission.

The guidance on the CDC website provides detailed information on how to practically accomplish the objectives of contact tracing. It outlines contact tracing preparation, implementation, and management to meet these objectives. Contact tracing preparation includes defining the roles and responsibilities within the contact tracing team, training personnel, and allocating funds and resources. Implementation of contact tracing includes identifying, listing, and enrolling persons as contacts, establishing contact follow-up processes, and discharging them after completion of monitoring. Management includes hiring and training of personnel, ensuring their health and safety, addressing stigma that might be associated with being a contact or contact tracing personnel, and establishing quality assurance measures (e.g., weekly active surveillance reports).

As the current Ebola outbreak continues, this document provides countries without Ebola outbreaks with guidance on preparing, implementing, and managing contact tracing to stop secondary Ebola transmissions in the event of an imported case. Among other public health measures, prompt and efficient contact tracing is crucial to terminate the transmission of Ebola.

## American Heart Month — February 2015

February is American Heart Month. The leading cause of death in the United States continues to be cardiovascular disease (CVD), which includes heart disease, hypertension (high blood pressure), and stroke. Although the rate of death attributable to CVD is decreasing (1,2), too few U.S. adults exhibit measures of good cardiovascular health, including adequate physical activity, a healthy diet, and ideal blood pressure. Additionally, more than one in three U.S. adults have at least one type of CVD, and nearly one in three deaths are attributed to CVD (1).

CVD and its risk factors are not distributed evenly across the U.S. population. Certain groups, defined by age, sex, race, ethnicity, or geography, have higher levels than others (1). Disproportionately high rates of avoidable CVD deaths are found among black men and among adults aged 30–74 years living in the Southeast (3), highlighting the need for targeted efforts to alleviate disparities and improve health (4). Black men experience a death rate attributable to CVD that is about 2.7 times higher than that of the lowest rate, found among white women (4). The reduction of CVD disparities and CVD overall are goals CDC aims to achieve through increased use of clinical protocols (5), partnerships with national, state, and local organizations, and educating persons at risk for CVD.

In observance of American Heart Month 2015, CDC is focusing on increased targeted consumer and health care provider messaging, as well as providing resources specifically for black men. Additional information is available at http://www.cdc.gov/dhdsp/american_heart_month.htm and http://millionhearts.hhs.gov.
